# Brain Metabolisms Involved in Self-Reported Quality of Mobility in Parkinson’s Disease

**DOI:** 10.3389/fnins.2020.00715

**Published:** 2020-07-07

**Authors:** Lu Fei, Feng-Tao Liu, Yi-Qi Liu, Jing-Jie Ge, Jia-Ying Lu, Shu-Jin He, Yi-Min Sun, Jian-Jun Wu, Chuan-Tao Zuo, Jian Wang

**Affiliations:** ^1^Department of Neurology and National Clinical Research Center for Aging and Medicine, Huashan Hospital, Fudan University, Shanghai, China; ^2^Department of Neurology, Huashan Hospital North, Fudan University, Shanghai, China; ^3^PET Center and National Clinical Research Center for Aging and Medicine, Huashan Hospital, Fudan University, Shanghai, China; ^4^Human Phenome Institute, Fudan University, Shanghai, China; ^5^Institute of Functional and Molecular Medical Imaging, Fudan University, Shanghai, China

**Keywords:** Parkinson’s disease, quality of life, PET, dopamine transporter, glucose metabolism

## Abstract

**Background:**

Objective motor ratings and subjective motor complaints are both widely used in Parkinson’s disease (PD). However, the objective basis to the self-perceived mobility quality is still not well elucidated.

**Purposes:**

We aimed to figure out the relevancy between the UPDRS motor scores and PDQ39 mobility sub-scores, and further explore whether physician-assessed motor dysfunctions and patients-reported mobility deficits have some shared mechanisms.

**Methods:**

49 patients with PD who completed the PDQ39 scale were retrospectively included. The relevancy between mobility quality and UPDRS scores was assessed, as well as the related presynaptic dopaminergic binding (^11^C-CFT) and glucose metabolism (^18^F-FDG) in this dual-tracer PET imaging study.

**Results:**

Modest correlation was found between UPDRS motor score and the PDQ39 mobility sub-score (*r* = 0.440, *p* = 0.002). No correlation was found between PDQ39 mobility SI and the dopaminergic lesions in putamen; however, the strict correlation was found with the UPDRS motor scores. In terms of global PD related pattern (PDRP) scores, the two motor scores both correlated strictly. In the further regional metabolism exploration, cerebellum correlated positively with PDQ39 mobility sub-scores, and the frontal and parietal regions mainly correlated negatively with the motor quality scores.

**Conclusion:**

UPDRS motor scores and PDQ39 mobility scores were only modestly correlated. The mechanisms involved under mobility quality were beyond dopaminergic deficiency, including motor related cerebellum hyper-metabolism and non-motor related frontal hypo-metabolism. Conclusively, the self-reported mobility experience may have the neurophysiological basis related to both motor and non-motor manifestations in PD.

## Introduction

Parkinson’s disease is a most common neurodegenerative movement disorder, in which the motor evaluation is of great importance. In clinical practices, the motor assessment can be obtained by objective motor rating from physicians and subjective motor complaints from patients. The UPDRS is the most commonly used scales by physicians for motor evaluation (Goetz et al., 2008). The 39-item Parkinson’s Disease Questionnaire is the most commonly adopted patient completed rating scale in PD QoL evaluation (Marinus et al., 2002), in which mobility is one of eight subdomains. Such assessments are quite helpful for disease severity assessment and therapeutic evaluation.

Though higher UPDRS motor scores contribute to poor QoL in PD patients, the improvement of motor symptoms does not necessarily guarantee better subjective feeling. In our previous study (Liu et al., 2019) including 45 PD patients with 1-year follow-up after DBS, the motor severities were greatly improved, however, only half participants were satisfied with the QoL improvement. Similarly, in a study (Daniels et al., 2011) including 61 PD patients after bilateral STN-DBS surgery, 57.0% of them did feel improved about their QoL despite 43.0% patients didn’t show satisfaction. Therefore, there should be similarities and differences between the subjective and objective motor assessments.

Objective motor evaluation may have some correlation with subjective motor experience, and such correlation should be fixed on some common material base. PET imaging has proven useful to explore the underlying mechanisms for clinical phenomenon of PD. ^11^C-CFT PET imaging could reflect the presynaptic DAT dysfunction (Nandhagopal et al., 2009), and ^18^F-fluorodeoxyglucose (^18^F-FDG) PET has been used for measuring the metabolic abnormalities in PD patients. Our previous findings revealed the relationship between clinical manifestations, dopaminergic and glucose-metabolic PET imaging in a dual-tracer PET imaging cohort (Liu et al., 2018). However, until now almost no study pays attention to the subjective bias of individual feelings related to the motor symptoms, and PET imaging may be helpful to understand the neurobiological basis in the patients’ subjective bias to the motor deficits in PD.

In our retrospective study, we aimed to figure out the relevancy between UPDRS motor score and QoL mobility subdomain, the two motor evaluation methods in PD. Furthermore, we attempted to figure out whether physician-assessed and patients-reported mobility dysfunctions had some shared material basis via cerebral presynaptic dopaminergic and glucose metabolic characteristics in our dual-tracer PET imaging cohort.

## Subjects and Methods

### Subjects

We retrospectively included 49 patients (31 males/18 females, 53.43 ± 12.06 years old) who completed the QoL evaluation (PDQ39 scale) from our previous DTPD cohort (Liu et al., 2018). DTPD study was a dual-tracer PET imaging study in Parkinson’s disease ever performed in Huashan Hospital between January 2010 and June 2014 (Liu et al., 2018). In the DTPD cohort, 103 patients (65 males/38 females, 54.1 ± 12.0 years old) diagnosed as PD were scanned with both ^11^C-CFT and ^18^F-FDG PET. All subjects have written informed consent according to the Declaration of Helsinki. The study was approved by the Human Studies Institutional Review Board, Huashan Hospital, Fudan University.

### Study Design

This is a retrospective study. The 49 PD subjects completed the PDQ39 scale, the UPDRS and H&Y scale in the “off” state. After the clinical assessments, all subjects were scanned with ^11^C-CFT PET and ^18^F-FDG PET.

### PET Imaging and Imaging Processing

Before the scanning, all the subjects withdrew anti-parkinsonian drugs for at least 12 h and started the fast for 6 h. Before the injection and PET imaging, all the participants should be settled under dark and quiet environment and stay sober during the whole examination. The equipment used for PET imaging in our center is Siemens’ Biograph 64 PET/CT scanner. During the scanning, cranial computed tomography (CT) was firstly conducted for photon attenuation correction.

In ^11^C-CFT PET imaging, 60 to 80 min after the intravenous injection of CFT (350–400 MBq), scanning data were obtained and then reconstructed with the OSEM method. In ^18^F-FDG PET imaging, within 45 to 55 min post-injection (150–200 MBq), scanning data were obtained for 10 min and then reconstructed with the OSEM method. All the PET data was captured in a three-dimensional (3D) mode. ^18^F-FDG was used to calculate rCMRglc.

^11^C-CFT and ^18^F-FDG PET data were both reconstructed using SPM5 software (Statistical Parametric Mapping; Wellcome Department of Imaging Neuroscience, Institute of Neurology, London, United Kingdom) implemented in Matlab7.4.0 (MathWorks Inc., Sherborn, MA, United States). A brain DAT binding template in Montreal Neurological Institute (MNI) space that was created by using ^11^C-CFT PET and corresponding structural MR images of another group consisting of 16 normal controls was used to normalize the ^11^C-CFT images. The procedures were presented in detail in former studies (Bu et al., 2018). As for ^18^F-FDG images, template within the SPM software was used for normalization. The normalized PET images were smoothed by a Gaussian filter of 10 mm FWHM (Full Width at Half Maximum) over a 3D space to increase signal to noise ratio for statistical analysis.

### Quantitative Analysis of Imaging Processing

To realize the individualized quantitative analysis of ^11^C-CFT binding of every participant, we placed standardized ROIs for caudate, anterior putamen, posterior putamen and occipital cortex on the mean image summed over central slices and manually adjust their position to meet the requirement, and subsequently, confirm the individual data with reference to standardized cerebral template in SPM5. The standard uptake value ratio (SUVR) of regional ^11^C-CFT binding in striatum was calculated by (striatum -occipital)/occipital counts as described previously (Ma et al., 2002).

The analysis of PDRP was based on an independent Chinese cohort (Wu et al., 2013). PET data of every patient can be calculated with independent network value using voxel-based algorithm (ScAnVP software^1^; at the Centre for Neuroscience, the Feinstein Institute for Medical Research, Manhasset, NY, United States), and then transformed into *Z* value (*Z* score) using subject scores of the healthy controls in the Chinese derivation cohort for PDRP as described previously.

To investigate the relationship between whole-brain metabolism and the mobility sub-scores, a multiple regression analysis was performed in SPM5. The global metabolic values of individual patient were entered as covariates in the ANCOVA model. To evaluate the significant correlation, we set the voxel threshold at *P* < 0.001 over whole-brain and search.

### Statistics Analysis

Quantitative data was performed as Mean ± Standard Deviation (SD). Kolmogorov–Smirnov test was used to evaluate the normal distribution of the continuous variables, if the data in different groups meets the standard of normal distribution, *T*-test was applied to compare the variable differences of clinical information, PDRP Z score and SUVRs of regional DAT binding in striatum between PD-QoL group and PD-Non-QoL group, if not, Mann Whitney’s *U* test was employed. Pearson correlation analysis was performed to analyze the correlation among DAT binding (ROIs), PDRP value and the two motor ratings in all patients. All analyses were settled with SPSS 22.0 (SPSS Inc., Chicago, IL, United States), and the two tailed *P* < 0.05 was considered as significant.

## Results

The demographic and clinical information were demonstrated in Table 1, and there was no obvious difference between the 49 included PD patients with QoL assessment (PD-QoL patients) and those without QoL assessment (PD-Non-QoL patients) (*p* > 0.05). In both ^11^C-CFT PET imaging and ^18^F-FDG PET imaging, the two groups matched well with each other, with no significant differences (Table 1).

**TABLE 1:** Demographic and clinical characteristics (including PET information) in the PD patients.

	**DTPD-QoL**	**DTPD-non-QoL**
Gender (Male/Female)	31/18	34/20
Age at onset (year)	50.85 ± 12.66	52.65 ± 11.73
Age at examination (year)	53.43 ± 12.06	54.67 ± 12.10
Disease Duration (month)	26.00 (14.00, 49.75)	19.00 (11.50, 36.00)
Hoehn and Yahr	2.00 (1.00, 2.00)	2.00 (1.00, 2.00)
LEDD (mg/day)	300.00 (75.00, 400.00)	200.00 (0.00, 318.75)
**^11^C-CFT Imaging**		
Contralateral Caudate	1.20 ± 0.36	1.27 ± 0.34
Contralateral Ant Putamen	0.86 ± 0.23	0.96 ± 0.33
Contralateral Pos Putamen	0.50 ± 0.12	0.59 ± 0.25
Ipsilateral Caudate	1.36 ± 0.38	1.38 ± 0.34
Ipsilateral Ant Putamen	1.09 ± 0.33	1.18 ± 0.37
Ipsilateral Pos Putamen	0.65 ± 0.21	0.73 ± 0.29
**^18^F-FDG Imaging**		
PDRP	3.19 ± 1.17	3.13 ± 1.20

*Data are given as mean ± standard deviation (SD) values or median (interquartile range). DTPD, dual-tracer PET imaging of Parkinson’s disease. QoL, quality of life. LEDD, Levodopa equivalent daily dosage. UPDRS, Unified Parkinson’s Disease Rating Scale. Rar1, Rating of akinesia and rigidity.*

### Correlation Between the Physician-Assessed and Patients-Reported Mobility Deficits

**FIGURE 1:**
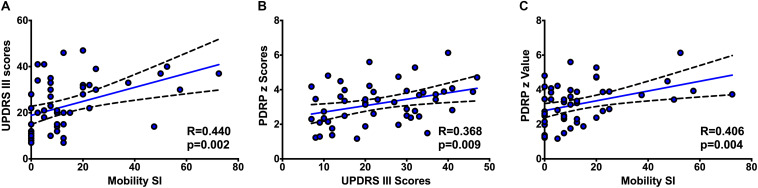
The correlations between UPDRS III scores and PDQ-39 mobility SI **(A)**, PDRP z scores and UPDRS III scores **(B)**, and PDRP *z* scores and PDQ-39 mobility SI **(C)**.

In the Pearson correlation analysis, a modest correlation between the UPDRS motor score and PDQ39 mobility SI was found (*r* = 0.440, *p* = 0.002) (Figure 1A), supporting the correlation between the physician-assessed and patients-reported mobility deficits.

### Correlations Between Striatal DAT Bindings (ROIs), UPDRS Motor Scores and PDQ39 Mobility SI

In our study, UPDRS III score correlated significantly (*p* < 0.01) with the striatal DAT binding (ROIs) (on the contralateral side, caudate: *r* = −0.462, *p* = 0.001; anterior putamen: *r* = −0.486, *p* = 0.000; posterior putamen: *r* = −0.419, *p* = 0.003; on the ipsilateral side, caudate: *r* = −0.450, *p* = 0.001; anterior putamen: *r* = −0.495, *p* < 0.001; posterior putamen: *r* = −0.409, *p* = 0.004). However, no significant correlation (*p* > 0.05) was found between the PDQ39 mobility SI and the DAT binding (ROIs) in putamen, but weak correlation was found in the caudate (on the contralateral side, caudate: *r* = −0.304, *p* = 0.033; anterior putamen: *r* = −0.202, *p* = 0.163; posterior putamen: *r* = −0.130, *p* = 0.374; on the ipsilateral side, caudate: *r* = −0.310, *p* = 0.030; anterior putamen: *r* = −0.258, *p* = 0.073; posterior putamen: *r* = −0.135, *p* = 0.357).

### Correlations Between Brain Glucose Metabolism, UPDRS III Scores and PDQ39 Mobility SI

In our study with ^18^F-FDG PET imaging, the PDRP z scores correlated positively with the UPDRS motor scores (*r* = 0.368, *p* = 0.009) (Figure 1B), as well as a positive interaction with PDQ39 mobility SI (*r* = 0.406, *p* = 0.004) (Figure 1C).

**FIGURE 2:**
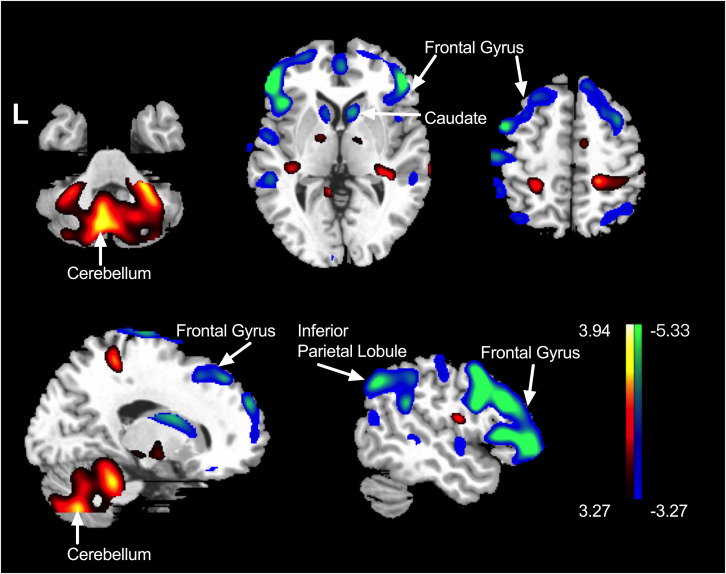
The brain regions correlating with the mobility sub-score. Positively correlated regions were displayed using a red–yellow scale and negatively correlated regions were displayed using a blue–green scale. The overlays are depicted in neurologic orientation. The gray-scale image is the standard T1-weighted structural magnetic resonance image (MRI) in Montreal Neurological Institute (MNI) space. The thresholds of the color bars represent *T* values. Voxel threshold was set at *P* < 0.001.

Besides the PDRP scores, we also explored the brain regions correlating with the PDQ39 mobility SI. As was shown in the Table 2 and Figure 2, cerebellum correlated positively with the PDQ39 mobility subdomain, and brain regions that correlated negatively with the mobility subdomain were mainly distributed in the caudate, inferior and middle frontal lobe and parietal lobe regions. The cerebral regions related to PDQ39 mobility SI was similar to the regions previously reported in the PD-related metabolic pattern.

**TABLE 2:** Brain regions exhibiting significant correlations between the PDQ-39 mobility sub-scores and regional brain metabolism.

**Regions**	**BA**	**MNI coordinates^a^**			**Zmax**	**Cluster Size (mm^3^)^b^**
		**x**	**y**	**Z**		
**Positive^b^**						
Rt Cerebellum Pyramis	/	8	–68	–36	3.94	8480
Lt Cerebellum Tonsil	/	–26	–42	–40	3.79	8480
Lt Cerebellum Culmen	/	–16	–42	–28	3.76	8480
Lt Cerebellum Inferior Semi-lunar Lobule	/	–16	–66	–48	3.71	1088
Lt Cerebellum Inferior Semi-lunar Lobule	/	–32	–64	–50	3.49	1088
**Negative^b^**						
Rt Inferior Frontal Gyrus	45	54	38	2	5.33	10584
Rt Middle Frontal Gyrus	6	52	4	48	5.03	10584
Rt Middle Frontal Gyrus	46	52	24	26	4.91	10584
Lt Middle Frontal Gyrus	46	–46	22	30	4.78	5800
Lt Inferior Frontal Gyrus	45	–48	38	4	4.41	5800
Lt Inferior Frontal Gyrus	47	–34	32	–14	4.23	5800
Rt Inferior Parietal Lobule	40	62	–46	44	4.6	3880
Rt Inferior Parietal Lobule	39	52	–64	42	4.21	3880
Rt Supramarginal Gyrus	40	56	–48	30	3.85	3880
Rt Superior Temporal Gyrus	42	68	–14	8	3.91	696
Lt Caudate	/	–12	10	10	3.88	872

*BA, Brodmann area; MNI, Montreal Neurological Institute; Lt, left; Rt, right; a, coordinates are displayed in Montreal Neurological Institute standard space. b, significant at voxel threshold of *p* < 0.001, extent threshold = 83 voxels (664 mm^3^).*

## Discussion

In this retrospective study, a modest correlation was found between UPDRS motor ratings and PDQ39 mobility sub-scores. The UPDRS motor scores correlated strictly with striatal dopaminergic lesions. However, the mechanisms related to PDQ39 mobility were beyond striatal dopaminergic deficiency. In the glucose metabolic analysis, the poor mobility quality was found to correlate with higher global PDRP scores, increased metabolism in cerebellum and decreased metabolism in prefrontal gyrus. Our study might offer a new perspective in understanding the objectively assessed and self-reported motor dysfunctions in PD.

As shown in our study, the UPDRS motor scores correlated modestly with the self-reported mobility dysfunction affecting the QoL. Although they both reflected the motor dysfunction, they lay emphasis on different aspects of motor deficits in PD. UPDRS motor scores record detailed motor descriptions and severities, nevertheless, PDQ39 is dedicated on the QoL of PD patients, more inclined to individual experience. Besides the different contents between the two scales, as aforementioned, subjective improvement of motor symptoms was not equivalent to better objective experience. Skorvanek et al. (2015) reported the overall burden of non-motor symptoms in PD was prior to motor symptoms with regard to QoL, and similar outcome was acquired that PDQ39 mobility sub-score was related to the MDS-UPDRS items pain and other sensations, fatigue and higher MDS-UPDRS part III scores.

Similar to our previous reports, the motor deficits as detected by UPDRS III scores correlated significantly with the dopaminergic lesions in putamen. To our surprise, the mobility QoL was irrelevant with the dopaminergic loss in putamen, but correlated more with specific cerebral glucose metabolism. This result inspired us that the perception of mobility dysfunction was beyond dopaminergic lesions.

The parkinsonian motor features could not be fully explained by dopamine depletion alone, and the brain energy metabolism might offer another perspective to understand the cause of motor symptoms. Schrack et al. (2010) performed a research on metabolic change with aging based on the BLSA and indicated that the changing movement performance may be an adaption to aging and neurodegeneration since available energy supplied for human physical needs in addition to RMR declined with aging. Some studies (Amano et al., 2015) further supported the hypothesis that neural metabolic dysfunction could play a role in Parkinsonian motor symptoms. In our previous dual-tracer PET study (Liu et al., 2018), the tremor severities did not correlate with striatal dopaminergic lesions, but correlated with the global PDRP values and related regional glucose metabolism. Therefore, the cerebral changes related to the motor lesions were beyond dopaminergic dysfunctions.

In our study, the metabolism in cerebellum correlated positively with PDQ39 mobility sub-scores, and the metabolism in inferior and middle frontal gyrus correlated negatively with mobility sub-scores. In detail, the higher PDQ39 mobility scores, indicating poorer movement performances, tended to be accompanied by elevated metabolism in cerebellum and decreased metabolism in frontal cortex.

The hyper-metabolism in cerebellum was a typical feature as suggested in the PDRP (Ma et al., 2007). In PD, the dopamine depletion in striatum consequently lead to dysfunctions in cortico–striatal–thalamic–cortical circuits (Alexander et al., 1986), inducing many functional compensation pathways including cerebellum (Wu and Hallett, 2005). Such compensatory effects by cerebellum might be mediated by the overlapping cortical areas between cerebello-thalamo-cortical circuits (Middleton and Strick, 2001) and BG circuits (Middleton and Strick, 2000), or direct projections from the cerebellum to the BG (Ichinohe et al., 2000; Hoshi et al., 2005). In our study, the increased metabolism in cerebellum accompanied poor mobility quality, supporting some neurophysiological basis to the self-perceived mobility feelings.

Besides cerebellum, the metabolism in prefrontal cortex was also found to be involved in mobility quality perception. As previously reported, decreased functional connectivity between these prefrontal regions and the putamen should be responsible for cognitive deficits in PD patients, and more severe cortical thinning in frontal and temporo-parietal cortices has been found in PD patients with mild cognitive deficits (Wilson et al., 2019). Also, an ^18^F-FDG-PET study (Wang et al., 2017) including 28 PD patients with anxiety has revealed decreased glucose metabolism in the bilateral orbitofrontal cortex. All these data supported the clinical findings that complex non-motor symptoms would interfere in the subjective experience in addition to motor symptoms. However, the underlying pathophysiological mechanism still remains to be defined.

## Conclusion

Though both of them were widely used to assess motor dysfunctions in PD, UPDRS motor scores, and PDQ39 mobility scores were not completely a same thing, showing only modest correlations. In this dual tracer PET imaging study, the mechanisms involved under mobility quality were beyond dopaminergic deficiency. The motor related cerebellum hyper-metabolism and non-motor related frontal hypo-metabolism contributed to the poor mobility quality. Conclusively, the self-reported mobility experience may have the neurophysiological basis related to both motor and non-motor manifestations in PD.

## Data Availability Statement

The raw data supporting the conclusions of this article will be made available by the authors, without undue reservation.

## Ethics Statement

The studies involving human participants were reviewed and approved by the Ethics Committee of Huashan Hospital, Fudan University. The patients/participants provided their written informed consent to participate in this study.

## Author Contributions

JW, C-TZ, J-JW, and F-TL conceived the research project. LF, F-TL, Y-QL, J-JG, and Y-MS organized the research project. LF, Y-QL, J-JG, J-YL, and F-TL executed the research project. JW and C-TZ designed the statistical analysis. S-JH, F-TL, Y-QL, LF, and J-YL executed the statistical analysis. JW, Y-MS, and F-TL reviewed and critiqued the statistical analysis. LF, F-TL, Y-QL, J-JG, and J-YL wrote the first draft of manuscript. JW, C-TZ, and F-TL reviewed and critiqued the manuscript. All authors contributed to the article and approved the submitted version.

## Conflict of Interest

The authors declare that the research was conducted in the absence of any commercial or financial relationships that could be construed as a potential conflict of interest.

## Footnotes

1 http://www.feinsteinneuroscience.org

## Abbreviations

^18^F-FDG: ^18^F-fluorodeoxyglucose
BG: basal ganglia
BLSA: Baltimore Longitudinal Study of Aging
DAT: dopamine transporter
DBS: deep brain stimulation
H&Y: Hoehn and Yahr
OSEM: ordered subset expectation maximization
PD: Parkinson’s disease
PDQ39: the 39-item Parkinson’s Disease Questionnaire
PDRP: Parkinson’s disease related pattern
PET: positron emission tomography
QoL: quality of life
rCMRglc: regional cerebral metabolic rate of glucose
RMR: resting metabolic rate
ROI: region of interest
STN: subthalamic nucleus
UPDRS: Unified Parkinson’s Disease Rating Scale.
